# Genetic Connectivity in a Cooperatively Breeding Carnivore Between Two Protected Areas

**DOI:** 10.1002/ece3.71420

**Published:** 2025-05-14

**Authors:** Ariana L. Cerreta, Jennifer R. Adams, Bridget L. Borg, Mathew S. Sorum, Lisette P. Waits, David E. Ausband

**Affiliations:** ^1^ Idaho Cooperative Fish and Wildlife Research Unit, Department of Fish and Wildlife Sciences University of Idaho Moscow Idaho USA; ^2^ Laboratory for Ecological, Evolutionary and Conservation Genetics, Department of Fish and Wildlife Sciences University of Idaho Moscow Idaho USA; ^3^ National Park Service Denali National Park and Preserve Denali Park Alaska USA; ^4^ National Park Service Yukon‐Charley Rivers National Preserve Fairbanks Alaska USA; ^5^ Department of Fish and Wildlife Sciences University of Idaho Moscow Idaho USA; ^6^ U.S. Geological Survey, Idaho Cooperative Fish and Wildlife Research Unit University of Idaho Moscow Idaho USA

**Keywords:** Alaska, *Canis lupus*, dispersal, microsatellites, pedigree reconstruction, wolf

## Abstract

Wildlife populations are increasingly threatened by human activities. Most studies, however, are often short in duration or do not encompass the large spatial extent necessary to measure the potential effects of human activities on population vital rates. Furthermore, the life history features of species with high fecundity and excellent dispersal capabilities can act as buffers against the potential negative effects of human activities on their populations. We used a 30‐year dataset of genetic samples from gray wolves ( *Canis lupus* ) in Alaska, USA, to examine genetic connectivity and diversity between National Park units separated by a region with recurrent human‐caused mortality. We found that the two protected populations were genetically similar and that dispersal events occurred between them even though they are > 450 km apart. We posit that intact ecosystems and a history of continuous distribution of wolves surrounding the affected regions likely maintained the genetic connectivity of wolves in the two protected areas.

## Graphical abstract

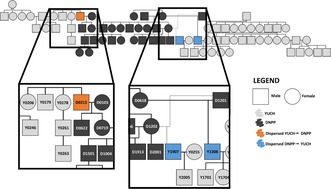

Genetic diversity, the variability in the genome present across individuals in a population, is an essential component of biodiversity for the conservation of wildlife populations (DeWoody et al. 2021; Hoban et al. 2022). As biodiversity and wildlife populations are increasingly threatened by human activities, such as overexploitation, habitat loss and fragmentation, and human‐mediated climate change (Maxwell et al. 2016; Scheffers et al. 2016), maintaining genetic diversity and connectivity can bolster the resiliency of a species to a rapidly changing environment via genetic adaptation (Frankel and Soulé 1981; Avise 2008). For the purposes of this article, we define genetic resiliency not only as evolutionary or adaptive potential (Forester et al. 2022), but also the ability of the population to maintain or recover genetic diversity and gene flow in the face of anthropogenic removal and harvest, climate change, and loss of habitat and landscape connectivity (Allendorf et al. 2022).

The gray wolf ( *Canis lupus* ) is a widely distributed, Holarctic, cooperatively breeding carnivore that has been variably managed and harvested throughout most of its range (Mech and Boitani 2003). Cooperatively breeding carnivores are typically composed of a pair of breeders and their non‐breeding offspring from previous years that assist in raising young of the year (Dickinson and Koenig 2016). In such cooperatively breeding carnivores, differing management strategies can not only impact the social composition of groups (Brainerd et al. 2008; Borg et al. 2015) but also the underlying genetic makeup of the population (Rick et al. 2017; Ausband and Waits 2020). Removal of individuals either by legal harvest, control or culling efforts, or illegal or accidental take (i.e., road mortalities) can disrupt social structure by reducing group size and/or removing breeders from the group (Brainerd et al. 2008; Borg et al. 2015).

Changes to group composition can alter the underlying genetic composition of the population and make the population susceptible to a loss of genetic diversity (Allendorf et al. 2013). However, removal may also increase opportunities for dispersers to mate, which may maintain or increase genetic diversity (Ausband and Waits 2020). In Minnesota, Rick et al. (2017) detected no change in heterozygosity (i.e., the proportion of individuals that have two different alleles at a locus and ranges from 0 to 1; Allendorf et al. 2022) or allelic richness (i.e., the number of variants present at a locus; Allendorf et al. 2022) in a local wolf population before and after a removal of approximately 24% of the population. Additionally, harvest rate (ranging from 0% to 27.6% over nine seasons of harvest) did not affect observed heterozygosity of individuals for wolves in Idaho (Ausband and Waits 2020). However, both studies found changes in other measures of genetic diversity after harvest was implemented, such as an increase in population structuring (i.e., individuals more genetically similar to members of the same subpopulation than to individuals outside of their subpopulation; Rick et al. 2017) and an increase in between‐group relatedness (Ausband and Waits 2020). Harvest has been shown to reduce effective migration (Rick et al. 2017) and may remove potential and actual dispersers from the population (Adams et al. 2008).

While most wolf populations in the United States were extirpated during the 19th and 20th centuries, robust wolf populations persisted across Canada and Alaska, USA (Stephenson et al. 1995; Boyd et al. 2023). In central Alaska, wolf populations are continuous, encompassing both public and private lands. Wolf harvest occurs throughout the state, including within some public lands administered by the National Park Service (NPS). Additionally, wolf control (i.e., lethal removal) efforts have been implemented in some areas with the goal of increasing ungulate populations (Titus 2007), potentially impacting the genetic connectivity of wolves throughout the state. Wolf social ecology in protected areas may also make the successful integration of dispersers into the population difficult. For example, wolves in protected areas typically exist at higher densities with reduced immigration and emigration rates (Jimenez et al. 2017; Sells et al. 2022). Thus, we examined genetic connectivity and diversity between two distant protected areas separated by a region with recurrent human‐caused mortality.

We evaluated population genetic structure between two protected areas, Denali National Park and Preserve (Denali) from 2004 to 2022 and Yukon‐Charley Rivers National Preserve (Yukon‐Charley) from 1993 to 2022. The lands located between these two areas were subject to a variety of management regimes over time, potentially affecting gene flow. Harvest or control efforts between the two protected areas remove individuals and create local vacancies that could be filled by remaining or newly immigrating wolves. Given that Denali and Yukon‐Charley are separated by 450 km and have shown geographic structuring in previous studies (Weckworth et al. 2011), albeit with smaller sample sizes, we hypothesized that there would be genetic differentiation (i.e., genetic structuring) between the two protected areas. Evidence for genetic differentiation would suggest that the wolf populations are genetically distinct in each area with little exchange of genetic material between them. However, the alternative hypothesis of no differentiation (i.e., genetic admixture) would suggest that the wolves belong to the same genetic population and have had sufficient gene flow over the study period to keep them connected. Understanding the genetic connectivity between these protected areas is important for managers to make informed decisions.

## 1 Methods

### 1.1 Study Area

**FIGURE 1:**
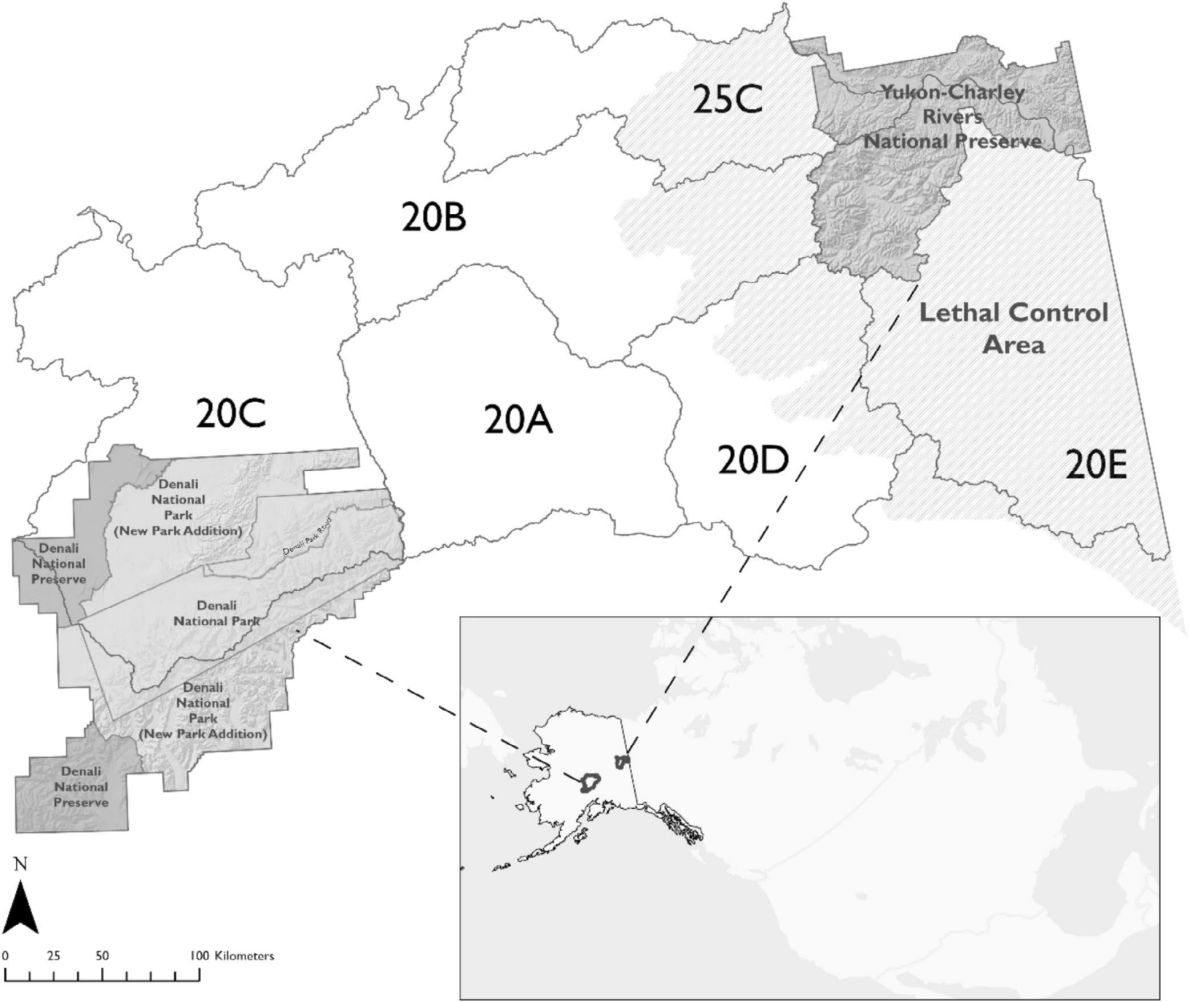
Locations of Denali National Park and Preserve and Yukon‐Charley Rivers National Preserve in Alaska, USA. Dark gray boxes adjacent to Denali indicate areas closed to harvest from 2000 to 2010. The region of light gray slanted stripes is the lethal control area adjacent to Yukon‐Charley. Game management units (20A–E, 25C) are represented in dark outlines and overlap portions of Denali and Yukon‐Charley.

Our study area consisted of two study sites, Denali (24,584 km^2^, Figure 1) and Yukon‐Charley (10,220 km^2^, Figure 1), in Alaska, USA. Both Denali and Yukon‐Charley were managed by the United States NPS. Denali, located approximately 230 km north of Anchorage, AK, was composed of a variety of land cover types such as high elevation alpine habitat and boreal forest (Borg et al. 2016). The southern portion of Denali was located south of the Alaska Range, resulting in a transitional maritime climate, whereas the northern region was characterized by an interior climate. Average annual precipitation in Denali was 40.4 cm (1981–2010) with an average annual total snowfall of 194.8 cm (1991–2020, National Park Service [NPS] 2022c). Elevation ranged from 68 to > 6100 m (NPS 2022a). In Denali, wolf hunting and trapping were allowed in the Preserve and additions outlined in the Alaska National Interest Lands Conservation Act (ANILCA, Public Law 96‐487, 94 Stat. 2371), but were not permitted in the original area of Denali (née Mt. McKinley) National Park. In management subunits adjacent to and overlapping Denali's northeast corner (GMU UCUs 605 and 607), an average of 4.7 (range 0–11) wolves were harvested annually from 2004 to 2022.

Yukon‐Charley Rivers National Preserve was in interior Alaska bordering Yukon Territory, Canada. The land cover was a mix of boreal forest and alpine habitat. Average annual precipitation was approximately 31.5 cm (1981–2010) in Yukon‐Charley (Eagle station) and snowpack peaked in March 2012 with an average depth of 51 cm (Coal Creek Climate Station; Sousanes and Hill 2014). Within the Preserve, elevations ranged from 183 to > 2000 m, with black spruce ( *Picea mariana* ) and deciduous hardwoods such as aspen ( *Populus tremuloides* ) and birch ( *Betula papyrifera* ) occurring at lower elevations (NPS 2022b; Schmidt et al. 2017). In Yukon‐Charley, sport and subsistence harvest of wolves was permitted throughout the Preserve, and approximately 12%–19% of wolves (of 75 and 68 collared wolves, respectively) were harvested by hunting or trapping (Schmidt et al. 2017). In addition, the Alaska Department of Fish and Game (ADF&G) implemented multiple wolf control efforts in the surrounding area over a 21‐year period beginning in 1997 that impacted wolf population dynamics within Yukon‐Charley (Boertje and Gardner 2003; Schmidt et al. 2017).

These surrounding areas and Game Management Units (GMUs; 20A, 20B, 20C, 20D, 20E, and 25C, Figure 1) located between the two protected areas were not included in our study area due to a lack of wolf genetic samples. However, to provide greater context for our study sites, it is important to note that wolves were distributed throughout the surrounding areas. Legal wolf harvest occurred throughout this area between Denali and Yukon‐Charley with ~210 wolves harvested annually across GMUs 20A, 20B, 20C, 20D, 20E, and 25C (Figure 1), totaling ~127,000 km^2^, an area larger than Pennsylvania (Young 2018; Schmidt and White 2018). Within the area, wolf density estimates varied between 4 and 22 wolves/1000 km^2^ (Young 2018; J. Wells, pers. comm.).

### 1.2 Field Methods

Sample collection occurred during ongoing wolf monitoring efforts in Denali from 2004 to 2022 and Yukon‐Charley from 1993 to 2022 (Figure S1). During these efforts, biologists captured and radio‐collared wolves within the parks under annual NPS permits and Institutional Animal Care and Use protocol approvals AKR_DENA_Borg_Wolves_2019.A3 and AKR_YUCH_Sorum_Wolf_2019.A3. Over the course of 35 years, biologists immobilized approximately 200 individual wolves from each park with 400–660 mg of Telazol (Tiletamine‐Zolazepam) hydrated to 5 mL with sterile water (Meier and Burch 2009). During handling, researchers collected and stored tissue, blood, and hair from individual wolves for future genetic analyses and disease screening. Detailed protocols can be found in Meier and Burch (2009).

### 1.3 Laboratory Methods

We extracted DNA from six sample types: (1) whole blood, (2) buccal swabs, (3) tissue, (4) hair, (5) blood clot, and (6) serum. Ages of samples varied greatly (see Tables S1–S4) and were extracted at the University of Idaho Laboratory for Ecological, Evolutionary and Conservation Genetics from 2019 to 2022. For buccal swabs, tissue, hair samples, and most whole blood, we extracted DNA using Qiagen DNeasy Blood and Tissue kits (Qiagen Inc., Valencia, CA, USA) according to manufacturer protocol. For samples with large swab tips, we added enough ATL reagent to cover the entire tip and scaled up the corresponding AL reagent and ethanol volumes to maintain equivalent proportions. All hair samples were extracted in a room designated for low‐quantity DNA. When possible, we attempted to use 10 follicles for each hair sample (Mumma et al. 2015). Blood clot, serum, and some whole blood samples were extracted using an in‐house protocol (Methods S1) with the Gentra Puregene Blood kit (Qiagen Inc., Valencia, CA, USA). We used negative controls for each extraction and all sample types to detect any potential contamination.

We genotyped samples with 2 different polymerase‐chain reaction (PCR) multiplexes consisting of a total of 18 dye‐labeled nuclear microsatellite loci and 2 sex identification loci (Clendenin et al. 2020; concentration and cycling in Tables S5 and S6). We amplified all whole blood, buccal swabs, tissue, and hair samples twice, and samples with a 40% amplification success rate after two PCRs remained in the dataset. We ran additional PCRs for samples where we detected allelic dropout. All blood clot and serum samples were amplified three times due to variable amplification success before discarding samples with insufficient loci amplified.

We separated PCR products by size using an Applied Biosystems 3130 × 1 capillary machine (Applied Biosystems Inc., Foster City, CA, USA) and called genotype peaks by eye with GENEMAPPER 5.0 (Applied Biosystems Inc.). For whole blood, buccal swabs, tissue, and hair samples, we required two independent PCRs to call genotypes and, when necessary, ran 1–3 additional PCR amplifications to resolve inconsistencies (Clendenin et al. 2020). Heterozygote calls required a consensus of ≥ 2 independent PCR amplifications at each locus for all sample types. Homozygote calls required ≥ 2 independent PCR amplifications at each locus for whole blood, buccal swabs, tissue, and hair samples but required an additional PCR amplification (≥ 3) for blood clot and serum samples due to the lower DNA quality of these samples. Only samples with > 60% complete loci were included in analyses. Overall, only five samples with < 90% complete loci were retained for analyses.

### 1.4 Analyses

For each study area and for the entire dataset, we evaluated whether each locus was in Hardy–Weinberg equilibrium using both *χ* ^2^ and exact tests (Guo and Thompson 1992) with the package “pegas” (Paradis 2010) in R version 4.2.2 (R Core Team 2021). We used Genepop 4.7.5 (Raymond and Rousset 1995; Rousset 2008) to assess genotypic linkage disequilibrium for each pair of loci for all individuals using Fisher's method. For each study area and the entire dataset, we calculated overall observed heterozygosity across loci by averaging the observed heterozygosity per locus using the package “hierfstat” (Goudet 2005).

To evaluate population genetic structuring, we performed a principal components analysis (PCA) using the package “ade4” (Thioulouse et al. 2018) in R. We also ran models for 1–10 clusters (i.e., Ks) with a burn‐in of 100,000 and MCMC repetitions of 100,000 for 10 iterations per K in the program STRUCTURE 2.3.4 (Pritchard et al. 2000) under the admixture model and assuming allele frequencies are correlated between populations. Using these results, we identified the most likely clustering number (i.e., K, the number of genetically distinct populations) by evaluating the likelihood curve and implementing the Evanno method (Evanno et al. 2005) using “pophelper” (Francis 2017).

To identify direct dispersal events and subsequent reproduction, we constructed pedigrees using Program COLONY v2.0.6.8 using maximum likelihood (Jones and Wang 2010). COLONY analyses included all individuals from both study areas and were run for five iterations. Considering first‐ and second‐degree relationship probabilities > 0.85 to construct pedigrees, we used capture and monitoring data to cross‐reference resulting pedigrees and to identify and remove any impossible relationships (e.g., a wolf that was a yearling in 2020 cannot be the parent of a wolf collared in 2009). We used information on group affiliation, capture location, years present in population, and the COLONY output to identify dispersal events followed by reproduction. For example, wolf Y1907 was collared in Yukon‐Charley but had a probability of 1.0 of being the offspring of two Denali collared wolves (see Figure 5, D1201 and D1202) based on their genotypes. Thus, we assumed that wolf Y1907 was born in Denali and then dispersed to Yukon‐Charley where it was later collared. Subsequently, we were able to determine that wolf Y1907 reproduced as it was assigned as the father to Y2005 with a probability of 0.9998. As wolf Y2005 was estimated to have been born in 2016 and wolf Y1907 was estimated to have been born in 2012 based on capture data, we concluded that the paternity assignment aligned with the years these wolves were known to be present in the population. Using all wolves from both study areas, we calculated *F*_st_ (Nei 1987) between the study areas with the package “hierfstat” (Goudet 2005) to evaluate gene flow. *F*_st_ was defined as

$$F_{\mathrm{st}}=\frac{\left(H_{t}-H_{s}\right)}{H_{t}},$$

where *H*_s_ is gene diversity within a population (Nei 1987; Goudet 2005).

$$H_{s}=\tilde{n}/\left(\tilde{n}-1\right)\left[1-\sum_{i}\bar{p_{i}^{2}}-H_{o}/2\tilde{n}\right],$$

where $H_{o}=1-\sum_{k}\sum_{i}P_{\mathrm{kii}}/\mathrm{np}$, *P*_kii_ is defined as the proportion of homozygotes *i* in sample *k*, np is the number of samples, $\tilde{n}=\mathrm{np}/\sum_{k}1/n_{k}$, and $\bar{p_{i}^{2}}=\sum_{k}p_{\mathrm{ki}}^{2}/\mathrm{np}$ (Nei 1987; Goudet 2005).

*H*_t_ was defined as

$$H_{t}=1-\sum_{i}{\overset{-}{p_{i}}}^{2}+H_{s}/\left(\tilde{n}\mathrm{np}\right)-H_{o}/\left(2\tilde{n}\mathrm{np}\right),$$

where $\bar{p_{i}}=\sum_{k}p_{\mathrm{ki}}/\mathrm{np}$. Thus, *F*_st_ measures how different a subgroup is from the entire population with values closer to 0 representing little differentiation (thus, high gene flow) and values closer to 1 representing high differentiation (thus, little gene flow) (Nei 1987; Goudet 2005).

## 2 Results

**FIGURE 2:**
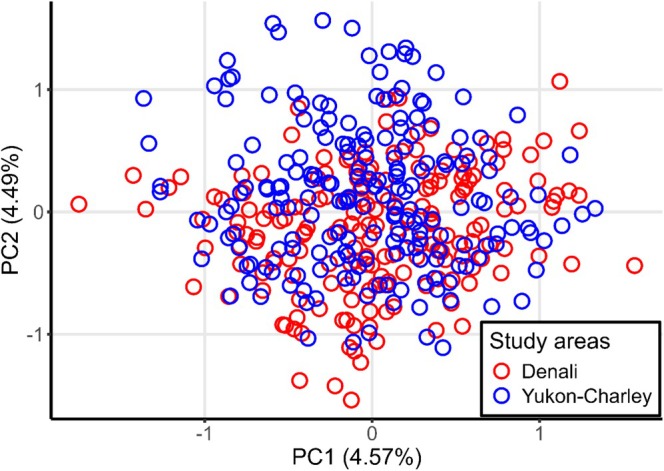
A graph of principal components 1 and 2 from the principal component analysis conducted using all wolf genotypes (*n* = 381) of 18 microsatellite loci from both Denali National Park and Preserve (Denali, 1998–2022, in red) and Yukon‐Charley Rivers National Preserve (Yukon‐Charley, 1990–2022, in blue).

**FIGURE 3:**
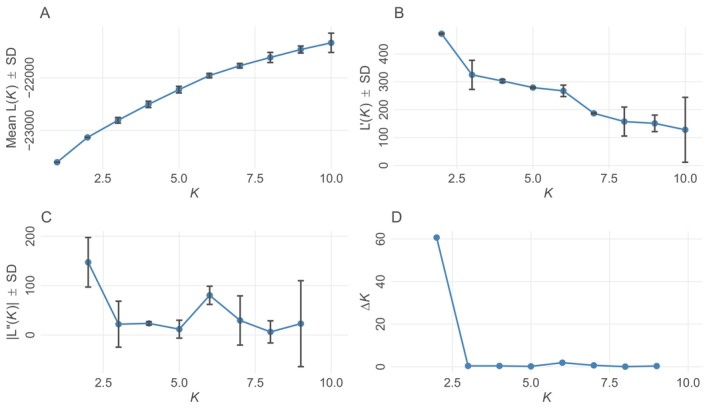
STRUCTURE analysis likelihood curve and Evanno method plots from “pophelper” (Evanno et al. 2005; Francis 2017) for 18 microsatellite loci generated for Denali (*n* = 183) and Yukon‐Charley (*n* = 198) gray wolves. Depicted are (A) mean *L*(*K*) for each *K* value; (B) rate of change of the likelihood distribution; (C) absolute values of the second order rate of change of the likelihood distribution; (D) *ΔK* = *m*|*L*″(*K*)|/*s*[*L*(*K*)] as defined in Evanno et al. (2005).

**FIGURE 4:**
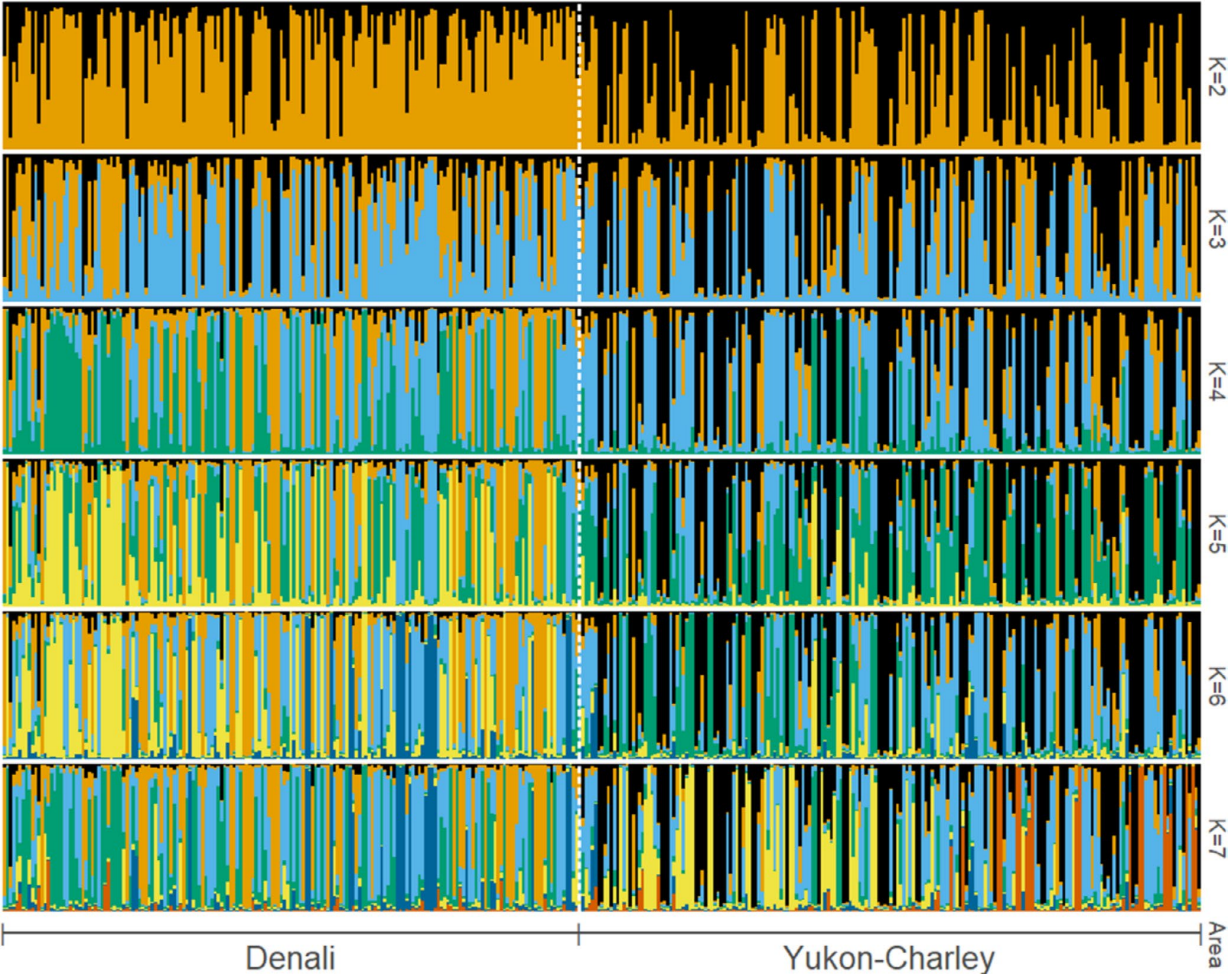
STRUCTURE plots for assumed population clusters, *K*, for 2–7. Each bar represents one individual and displays the proportion of their genotype that can be attributed to a genetic cluster (different colors). Considerable admixture can be seen for all values of *K*.

We genotyped 381 unique wolves (Denali, *n* = 183; Yukon‐Charley, *n* = 198) that were estimated to be present in the population from 1998 to 2022 in Denali and 1990 to 2022 in Yukon‐Charley. We genotyped an average number of 17.86 loci (out of 18 loci; mean genotype completeness of 99.2%) for the entire dataset and 17.83 and 17.89 loci for Denali and Yukon‐Charley, respectively. As expected in populations where mating is not random and only a few individuals reproduce in each group, *χ* ^2^ and exact tests suggested that some loci may not be in Hardy–Weinberg equilibrium (Tables S7–S9). The overall observed heterozygosity was 0.74 for the entire dataset, 0.72 for Denali, and 0.75 for Yukon‐Charley (Tables S7–S9). Additionally, Fisher's method to test linkage disequilibrium suggested that several loci may be out of linkage equilibrium (Table S10). We detected 10 and 21 private alleles (i.e., variations of a locus exclusive to a group or subpopulation) for Denali and Yukon‐Charley, respectively (Table S11). PCA showed substantial overlap of individuals from each sampling area along both PC1 and PC2, indicating a high degree of gene flow between wolves from Denali and Yukon‐Charley and no clear differentiation between sampling areas (Figure 2). The likelihood curve from STRUCTURE increased from *K* = 1 to *K* = 10 and the Evanno method supported *K* = 2 (or two clusters; Figure 3). The STRUCTURE plots revealed considerable admixture between the two clusters; however, the proportion of membership to each inferred cluster differed. In Denali, the average proportion of membership assigned to cluster 1 was 0.744 (represented by orange in Figure 4). In Yukon‐Charley, the average proportion of membership assigned to cluster 2 was 0.618 (represented by black in Figure 4). The PCA showed substantial overlap, and the STRUCTURE plots revealed considerable admixture as well. These results suggest that wolves from the two study areas belong to the same genetic population, although there may have been some restriction in gene flow, as evidenced by the average proportion of membership assigned to different clusters in the STRUCTURE analysis and the identification of private alleles.

**FIGURE 5:**
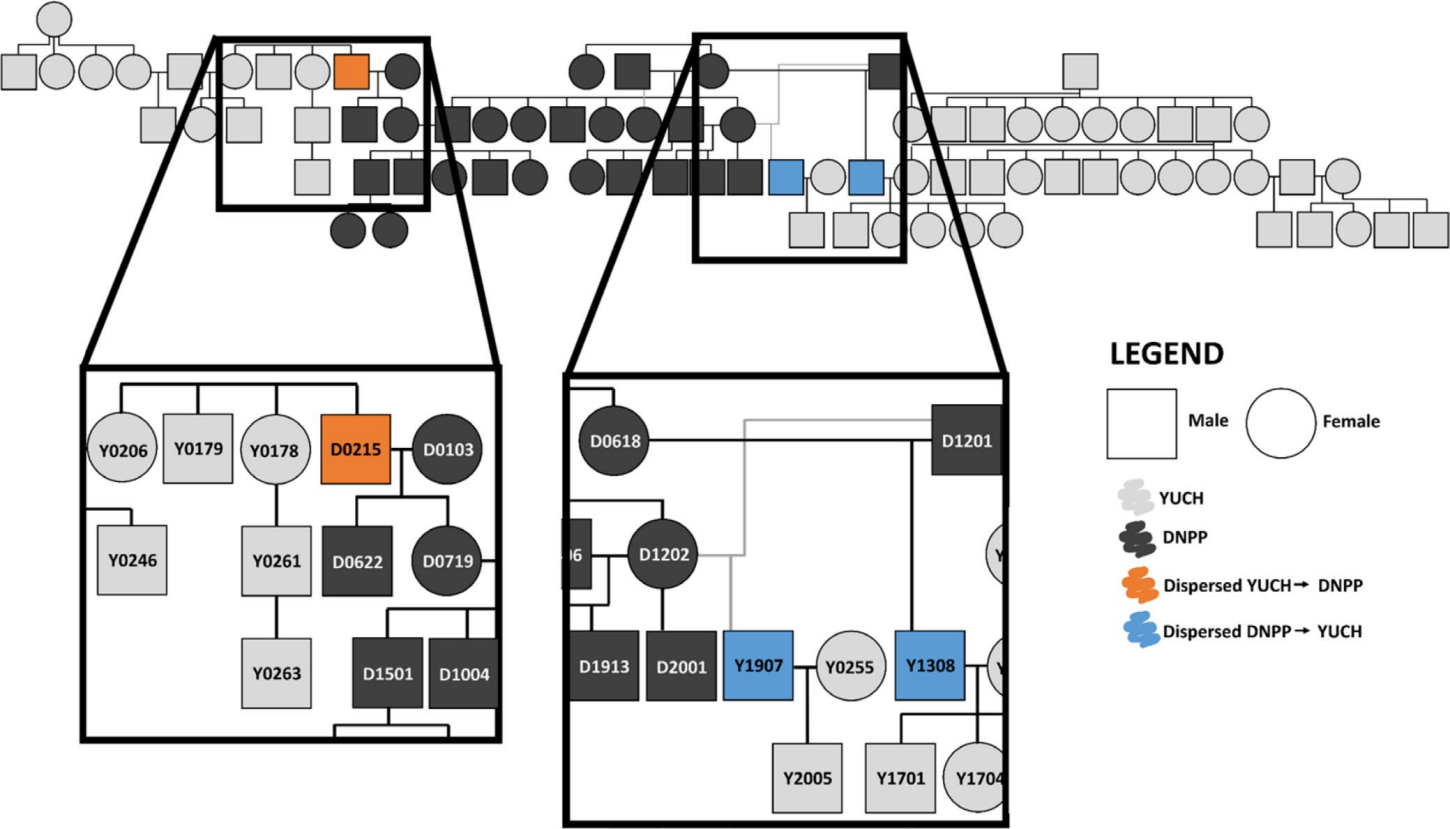
Pedigree of largest genetically related group, highlighting reproduction after a dispersal event. Light gray individuals originated in Yukon‐Charley Rivers National Preserve (YUCH), whereas dark gray individuals came from Denali National Park and Preserve (DNPP). Circles represent females and squares represent males. Orange represents a dispersal from YUCH to DNPP, whereas blue is dispersal from DNPP into YUCH. Of note, we identified offspring of these dispersers within our samples, and these are reflected in the pedigree.

Pedigrees contained first‐ and second‐degree relationships with probabilities > 0.85, often resulting in pedigrees connecting wolves from several observed groups defined during capture and monitoring. With the pedigree construction from COLONY, we identified 36 pedigrees of genetically related individuals (18 Denali pedigrees, 17 Yukon‐Charley pedigrees, 1 mixed pedigree) with the largest genetically connected pedigree containing 79 wolves from both Denali (28 individuals) and Yukon‐Charley (51 individuals; Figure 5). Within the largest pedigree, we documented three instances of direct gene flow between the study areas. Specifically, all instances were a result of male dispersal and subsequent reproduction with two males dispersing from Denali into Yukon‐Charley and one male dispersing from Yukon‐Charley to Denali (Figure 5). We were unable to assign 43 (23%) Denali and 38 (19%) Yukon‐Charley wolves to any pedigree. Across all management periods, pairwise *F*_st_ was 0.0064 between Denali and Yukon‐Charley, providing additional support that wolves from the two study areas belonged to the same genetic population.

## 3 Discussion

We found that, in contrast to our expectations, genetic diversity and connectivity of wolves appear to be intact within and between Denali and Yukon‐Charley in Alaska. Results from the Evanno method, PCA, and pedigree analysis suggest that wolves from both areas belong to the same population. We also observed direct dispersal between the two areas, confirming that dispersal has and does occur, despite varying levels of human‐caused mortality on the intervening lands. Together, these findings suggest that the take (mortality) of wolves implemented on the intervening unprotected lands did not restrict gene flow between the two study areas.

In contrast to the lower 48 states of the United States, central Alaska has maintained wolf populations throughout a relatively unfragmented ecosystem, with relatively intact habitat providing a level of connectivity between Denali and Yukon‐Charley's borders (Boyd et al. 2023). Wolf harvest historically and currently occurs throughout this corridor and the Alaskan range. However, we initially expected that human‐caused mortality and the distance separating the two study areas may be sufficient to limit genetic exchange due to the previous findings of genetic structuring in Alaskan wolves (Weckworth et al. 2011) and structuring in other harvested populations (Rick et al. 2017; Clendenin et al. 2019).

Surprisingly, we found no evidence for genetic differentiation. Rather, our study showed results similar to previous studies conducted across the northern portion of North America. In an analysis of wolves across much of northern Canada and Alaska, wolves from central Alaska were clustered with (i.e., closely related to) wolves from the Yukon territory and portions of northern British Columbia (Carmichael et al. 2007). We think the central Alaskan population of wolves is genetically connected due to the ubiquitous distribution of wolves in the state and via individual wolf dispersal. Wolves inhabit the intervening land between the study areas, and wolves are harvested at rates (2 wolves/1000 km^2^) lower than thresholds that have been reported to negatively impact population dynamics (< 29%, Adams et al. 2008). While this region is a spatial gap in our dataset, we suspect that genetic data from this region would further reduce the limited differentiation between our study sites or potentially reveal a gradient between them.

Wolves have a remarkable ability to disperse distances > 450 km, the distance separating our study areas. For example, wolves have been documented to disperse as much as 696 km from Denali (Mech et al. 1998) and 705 km from Yukon‐Charley (Schmidt et al. 2017). Additionally, successful dispersers may have taken advantage of territory openings in and around Yukon‐Charley created by predator control efforts. The two instances of direct dispersal from Denali to Yukon‐Charley documented by our study occurred during the predator control period and, in both instances, dispersers successfully reproduced in territories of groups that had been previously removed by predator control efforts. The presence of male dispersal between the two areas and subsequent reproduction demonstrates that gene flow can and does occur directly between our study areas. However, it is important to note that due to the lack of sampling in the region between the two study areas, we were likely unable to detect other dispersers moving into and out of Denali and Yukon‐Charley.

We found that measures of genetic diversity were comparable to both harvested and largely unharvested wolf populations. We observed overall heterozygosity values ranging from 0.72 in Denali to 0.75 in Yukon‐Charley (0.74, collectively), which are similar to those found in other wolf populations in western North America with comparable microsatellite data (largely unharvested, Yellowstone National Park (8991 km^2^), 0.73; vonHoldt et al. 2008; across three harvested study areas (10,438 km^2^) in Idaho, USA, 0.75; Ausband 2022). For more local comparisons, Weckworth et al. (2005) documented an observed heterozygosity of 0.59 (*n* = 29) for interior Alaska wolves and 0.69 (*n* = 14) for wolves from the Yukon Territory, Canada, across 12 nuclear microsatellite loci. These lower values were likely due to the use of fewer microsatellite loci and the fact that our 18 microsatellite loci were specifically chosen to have sufficient variation to identify sibling and family groups. In fact, Carmichael et al. (2007) reported an average expected heterozygosity (a different but related metric used for genetic diversity) of 0.73 (*n* = 322) for their Alaskan and western Yukon Territory wolf samples using 15 microsatellite loci. These heterozygosity values suggest that the wolves in our study areas had adequate genetic diversity to avoid inbreeding, thus contributing to the overall genetic resilience of the population. With sufficient heterozygosity, a population will be better able to recover or maintain genetic diversity when it encounters stressors such as harvest, climate, or loss of habitat (Allendorf et al. 2022).

Despite our finding of limited genetic differentiation between our study areas, changes in finer‐scale genetic metrics (i.e., individual or group‐based) may take more than a few generations to appear after a causative action interferes with the cooperative breeding of wolves. Wolf generation times typically range from 4.2 to 4.7 years (Mech and Barber‐Meyer 2017), thus our data spanned approximately five and seven generations for Denali and Yukon‐Charley, respectively. If changes to population structuring occurred after each generation, we would have been unable to detect them due to grouping samples across all years for our STRUCTURE analyses. Additionally, samples were unevenly distributed through time (Figure S1), making it difficult to evaluate population structure based on wolf generation times. As for finer‐scale metrics, harvest has been shown to decrease recruitment in wolf populations (Ausband et al. 2015, 2017) which decreases the number of potential future dispersers from a local area with direct impacts on gene flow. Alternatively, scenarios exist in which genetic diversity is maintained or increased as breeder death could increase opportunities for dispersers and unrelated individuals to mate (Ausband and Waits 2020).

Management actions and human‐caused mortality can potentially affect wolf social ecology and population demography even while genetic metrics in the populations remain unaffected. Group dynamics and persistence can vary depending on the individual removed (Brainerd et al. 2008; Borg et al. 2015). For example, the loss of breeders can affect the persistence of groups, with the loss of both breeders increasing the likelihood of group dissolution (Brainerd et al. 2008; Borg et al. 2015). Additionally, groups that lose only female breeders are more likely to dissolve than those that lose only male breeders (Borg et al. 2015). However, direct observation of pup survival, group dissolution, etc. solely using population‐level genetic metrics is difficult because knowledge about every individual within a group over time is necessary. As our samples originated from captures during collar deployment, we were unable to account for every individual within a group over consecutive years. Repeated sampling of every individual in a group, potentially through non‐invasive genetic sampling, could help provide the resolution of data necessary to examine these mechanisms in future studies (Stansbury et al. 2014). Even with incomplete sampling of wolf groups, however, our study demonstrated that these genetic data can still be useful for pedigree reconstruction and identification of dispersers using programs such as COLONY.

Genetic and genomic monitoring of wildlife populations, such as in this study, can provide insights for the effective management of protected areas, particularly when species within these areas may be affected by management activities outside their borders. For example, population monitoring can identify the first signs of inbreeding, track gene flow and genetic diversity, be used to examine group‐level dynamics, be beneficial for the evaluation of population recovery, and potentially aid genetic rescue efforts (Adams et al. 2011; Ausband et al. 2017). In all, our research suggested that large areas of habitat with relatively low levels of human fragmentation created a relatively intact ecosystem that allowed for genetic connectivity between the two protected areas despite ubiquitous wolf harvest and predator control programs in the intervening area.

## Author Contributions

**Ariana L. Cerreta:** data curation (equal), formal analysis (lead), investigation (lead), visualization (lead), writing – original draft (lead). **Jennifer R. Adams:** data curation (equal), formal analysis (equal), methodology (lead), supervision (supporting), validation (lead), writing – review and editing (equal). **Bridget L. Borg:** conceptualization (equal), data curation (equal), funding acquisition (equal), resources (equal), visualization (supporting), writing – review and editing (equal). **Mathew S. Sorum:** conceptualization (equal), data curation (equal), funding acquisition (equal), resources (equal), writing – review and editing (equal). **Lisette P. Waits:** supervision (supporting), writing – review and editing (equal). **David E. Ausband:** conceptualization (equal), funding acquisition (equal), resources (equal), supervision (lead), writing – original draft (supporting).

## Ethics Statement

Research was conducted under annual National Park Service permits and Institutional Animal Care and Use protocol approvals AKR_DENA_Borg_Wolves_2019.A3 and AKR_YUCH_Sorum_Wolf_2019.A3.

## Conflicts of Interest

The authors declare no conflicts of interest. This project was funded by the National Park Service and the United States Geological Survey. Any use of trade, firm, or product names is for descriptive purposes only and does not imply endorsement by the U.S. Government.

## Supporting information


**Table S1:** Percent success by sample year and type from Yukon‐Charley Rivers National Preserve. A successful sample led to a usable genotype (> 60% complete loci) throughout the amplification process. Samples were counted as failed if it was discarded due to insufficient amplification (< 40% amplification success rate) during the first two rounds of polymerase chain reaction amplification. Parenthetical values refer to sample size, *N*, for a given year and sample type.
**Table S2:** Continued percent success by sample year and sample type from Yukon‐Charley Rivers National Preserve. The final column refers to the total percent success by sample type across collection years. Parenthetical values refer to sample size, *N*, for a given year and sample type.
**Table S3:** Percent success by sample year and type from Denali National Park and Preserve. A successful sample led to a usable genotype (> 60% complete loci) throughout the amplification process. Samples were counted as failed if it was discarded due to insufficient amplification (< 40% amplification success rate) during the first two rounds of polymerase chain reaction amplification. Parenthetical values refer to sample size, *N*, for a given year and sample type.
**Table S4:** Continued percent success by sample year and sample type from Yukon‐Charley Rivers National Preserve. The final column refers to the total percent success by sample type across collection years. Parenthetical values refer to sample size, *N*, for a given year and sample type.
**Table S5:** Eighteen nuclear DNA microsatellite loci and two sex loci used for gray wolf identification, sibship, and sex confirmation. Loci were dye‐labeled and divided into two polymerase‐chain reaction (PCR) multiplexes with the listed volumes. Loci were selected from Holmes et al. (1994), Breen et al. (2001), Guyon et al. (2003), Salim et al. (2007), Ostrander et al. (2017) and PCR multiplexes from Clendenin et al. (2020), with modifications to primer input volumes. F+R indicates a mixture of forward and reverse primers.
**Table S6:** Thermocycler conditions for two polymerase‐chain reaction multiplexes used for gray wolf identification, sibship, and sex confirmation.
**Table S7:** Number of alleles, observed (*H*_o_), and expected heterozygosity (*H*_s_) by locus for all individuals (*N* = 381) sampled from Denali National Park and Preserve and Yukon‐Charley Rivers National Preserve. Additionally, results by locus are reported for the *χ* ^2^ and exact tests to evaluate Hardy–Weinberg equilibrium. *p*‐values for the *χ* ^2^‐test are reported in *p*‐value (*χ* ^2^), whereas *p*‐values from the exact test are under *p*‐value (exact).
**Table S8:** Number of alleles, observed (*H*_o_), and expected heterozygosity (*H*_s_) by locus for all individuals from Denali National Park and Preserve (*N* = 183). Additionally, results by locus are reported for the *χ* ^2^ and exact tests to evaluate Hardy–Weinberg equilibrium. *p*‐values for the *χ* ^2^‐test are reported in *p*‐value (*χ* ^2^), whereas *p*‐values from the exact test are under *p*‐value (exact).
**Table S9:** Number of alleles, observed (*H*_o_), and expected heterozygosity (*H*_s_) by locus for all individuals from Yukon‐Charley Rivers National Preserve (*N* = 198). Additionally, results by locus are reported for the *χ* ^2^ and exact tests to evaluate Hardy–Weinberg equilibrium. *p*‐values for the *χ* ^2^‐test are reported in *p*‐value (*χ* ^2^), whereas *p*‐values from the exact test are under *p*‐value (exact).
**Table S10:** *p*‐values from Fisher’s method to test genotypic linkage disequilibrium in Genepop 4.7.5 (Raymond and Rousset 1995; Rousset 2008).
**Table S11:** Private alleles detected from each study area by locus.
**Figure S1:** A histogram of the number of wolf samples that lead to successful individual genotypes (*n* = 381) by collection year used in analyses for this paper. Samples collected in Denali are red while samples collected in Yukon‐Charley are teal. These counts encompass all sample types, including whole blood, blood clot, serum, hair, tissue, and cheek swabs.

## Data Availability

The data that support the findings of this study are openly available in the Integrated Resource Management Applications (IRMA; a data repository for the U.S. National Park Service) at https://doi.org/10.57830/2308717.
