# Supplementary material for: Genetic Connectivity in a Cooperatively Breeding Carnivore Between Two Protected Areas
Source: Ecol Evol. 2025 May 14;15(5):e71420. doi: 10.1002/ece3.71420 (PMC12076063; doi:10.1002/ece3.71420)
Supplement: Supplementary file 1 — Table S1: Percent success by sample year and type from Yukon‐Charley Rivers National Preserve. A successful sample led to a usable genotype (> 60% complete loci) throughout the amplification process. Samples were counted as failed if it was discarded due to insufficient amplification (< 40% amplification success rate) during the first two rounds of polymerase chain reaction amplification. Parenthetical values refer to sample size, N, for a given year and sample type. Table S2: Continued percent success by sample year and sample type from Yukon‐Charley Rivers National Preserve. The final column refers to the total percent success by sample type across collection years. Parenthetical values refer to sample size, N, for a given year and sample type. Table S3: Percent success by sample year and type from Denali National Park and Preserve. A successful sample led to a usable genotype (> 60% complete loci) throughout the amplification process. Samples were counted as failed if it was discarded due to insufficient amplification (< 40% amplification success rate) during the first two rounds of polymerase chain reaction amplification. Parenthetical values refer to sample size, N, for a given year and sample type. Table S4: Continued percent success by sample year and sample type from Yukon‐Charley Rivers National Preserve. The final column refers to the total percent success by sample type across collection years. Parenthetical values refer to sample size, N, for a given year and sample type. Table S5: Eighteen nuclear DNA microsatellite loci and two sex loci used for gray wolf identification, sibship, and sex confirmation. Loci were dye‐labeled and divided into two polymerase‐chain reaction (PCR) multiplexes with the listed volumes. Loci were selected from Holmes et al. (1994), Breen et al. (2001), Guyon et al. (2003), Salim et al. (2007), Ostrander et al. (2017) and PCR multiplexes from Clendenin et al. (2020), with modifications to primer input volumes. F+R indicates a mi [file ECE3-15-e71420-s001.docx]

**Supplementary Information**

Article: Genetic Connectivity in a Cooperatively-breeding Carnivore between Two Protected Areas

Cerreta et al., 2025

Corresponding author: Ariana L. Cerreta, *Idaho Cooperative Fish and Wildlife Research Unit, Department of Fish and Wildlife Sciences, University of Idaho, 875 Perimeter Drive MS 1136, Moscow, ID 83844, USA, acerreta@uidaho.edu*

Supplementary Methods

Methods S1:

Blood Clot Extraction Protocol

Laboratory for Ecological, Evolutionary and Conservation Genetics, University of Idaho

Developed by Dorah Mtui and Jen Adams in consultation with Qiagen Inc. (Valencia, CA, USA)

We extracted DNA from whole blood, blood clot, and serum samples using the Gentra® Puregene® Blood Kit (Qiagen Inc., Valencia, CA, USA) using a three-day protocol. On the first day, cells were lysed from clotted blood, serum, or whole blood. We mixed 100 μl of the sample with 1100 μl Cell Lysis Solution and then added 6 μl Puregene® Proteinase K (20mg/ml). Samples were mixed by inversion and incubated at 55°C overnight.

The following day, we added 6 μl of RNase A Solution to the cell lysate, mixed by inversion, and incubated the samples at 37°C for 15 minutes. After incubation, samples were cooled to room temperature by placing them on ice for 1 minute. We then added 400 μl Protein Precipitation Solution, vortexed samples, and incubated on ice for 5 minutes. Following incubation, we centrifuged samples at 16,000 x *g* for 3 minutes. We added the supernatant from the sample to a mixture of 1200 μl of isopropanol and 2 μl of Glycogen Solution (20mg/ml). We mixed by inversion, centrifuged the supernatant mixture at 5000 rpm for 3 minutes, and carefully discarded the supernatant, leaving a DNA pellet at the base of the tube. We washed the DNA with 1200 μl of 70% ethanol via inversion and then centrifuged again at 5000 rpm for 3 minutes. We discarded the supernatant (ethanol) and left the DNA to air dry at room temperature for 15-30 minutes. After drying, we added 40 μl of DNA Hydration Solution and incubated the samples at 65°C for 1 hour. After initial incubation, samples were left to incubate at room temperature overnight with gentle shaking.

On the third day, we transferred the DNA extraction to long-term storage tubes and stored them at -18°C.

Supplementary Tables

Table S1: Percent success by sample year and type from Yukon-Charley Rivers National Preserve. A successful sample led to a usable genotype (>60% complete loci) throughout the amplification process. Samples were counted as failed if it was discarded due to insufficient amplification (<40% amplification success rate) during the first two rounds of polymerase chain reaction amplification. Parenthetical values refer to sample size, N, for a given year and sample type.

|  | **Percent Success by Sample Year (N)** | | | | | | | | | | | | | |
| --- | --- | --- | --- | --- | --- | --- | --- | --- | --- | --- | --- | --- | --- | --- |
| **Sample Type** | *1993* | *1994* | *1995* | *1996* | *1997* | *1998* | *1999* | *2000* | *2001* | *2002* | *2003* | *2004* | *2005* | *2006* |
| Whole Blood | 100 (2) | 100 (1) | 100 (1) | 100 (11) | - | - | - | 100 (8) | - | - | 0 (1) | - | 0 (1) | 100 (1) |
| Blood Clot | 50 (2) | 100 (1) | - | 100 (6) | 100 (4) | 95 (19) | 100 (1) | 100 (1) | 100 (7) | - | - | 100 (1) | - | 94 (18) |
| Serum | 0 (2) | - | 0 (1) | - | - | - | 0 (1) | 0 (2) | 67 (3) | 0 (2) | 0 (2) | 0 (1) | 100 (1) | - |
| Hair | - | - | - | - | - | - | - | - | - | - | - | - | - | 100 (3) |
| Tissue | - | - | - | - | - | - | - | - | - | - | - | - | - | 100 (1) |
| Cheek Swab | - | - | - | - | - | - | - | - | - | - | - | - | - | - |

*Continued*

Table S2: Continued percent success by sample year and sample type from Yukon-Charley Rivers National Preserve. The final column refers to the total percent success by sample type across collection years. Parenthetical values refer to sample size, N, for a given year and sample type.

|  | **Percent Success by Sample Year (N)** | | | | | | | | | | | | |
| --- | --- | --- | --- | --- | --- | --- | --- | --- | --- | --- | --- | --- | --- |
| **Sample Type** | *2007* | *2008* | *2009* | *2010* | *2011* | *2012* | *2017* | *2018* | *2019* | *2020* | *2021* | *2022* | ***Total*** |
| Whole Blood | 100 (5) | 100 (8) | 100 (6) | 100 (10) | 100 (3) | 100 (1) | - | - | - | 100 (11) | - | - | **97 (70)** |
| Blood Clot | 100 (1) | - | - | 100 (1) | - | 100 (3) | - | - | - | - | - | - | **95 (65)** |
| Serum | 0 (1) | 0 (1) | - | - | - | - | - | - | - | - | - | - | **18 (17)** |
| Hair | 100 (1) | - | - | - | - | - | - | - | 100 (4) | - | - | 100 (1) | **100 (9)** |
| Tissue | - | - | - | - | - | - | - | - | - | - | - | - | **100 (1)** |
| Cheek Swab | - | - | - | - | 100 (2) | 100 (1) | 100 (7) | 100 (8) | 100 (14) | 100 (2) | 100 (12) | 100 (11) | **100 (57)** |

Table S3: Percent success by sample year and type from Denali National Park and Preserve. A successful sample led to a usable genotype (>60% complete loci) throughout the amplification process. Samples were counted as failed if it was discarded due to insufficient amplification (<40% amplification success rate) during the first two rounds of polymerase chain reaction amplification. Parenthetical values refer to sample size, N, for a given year and sample type.

|  | **Percent Success by Sample Year (N)** | | | | | | | | | | |
| --- | --- | --- | --- | --- | --- | --- | --- | --- | --- | --- | --- |
| **Sample Type** | *2004* | *2005* | *2006* | *2007* | *2008* | *2009* | *2010* | *2011* | *2012* | *2013* | *2014* |
| Whole Blood | - | - | - | - | 100 (1) | - | 100 (7) | 100 (11) | 100 (11) | 100 (5) | 100 (1) |
| Blood Clot | - | - | - | - | - | 100 (3) | - | - | - | - | - |
| Serum | 0 (1) | - | - | 0 (1) | 13* (8) | - | 0 (2) | - | - | - | - |
| Hair | - | 100 (1) | 95 (19) | 100 (21) | 100 (7) | 100 (9) | 100 (1) | - | - | - | - |
| Tissue | - | - | - | - | - | - | - | - | - | - | - |
| Cheek Swab | - | - | - | - | - | - | - | 100 (1) | - | - | - |
| *Only one individual (of 8) was successfully genotyped from serum this year. The successful sample had to be extracted twice to get sufficient loci due to allelic dropout with the first extraction. | | | | | | | | | | |  |

*Continued*

Table S4: Continued percent success by sample year and sample type from Yukon-Charley Rivers National Preserve. The final column refers to the total percent success by sample type across collection years. Parenthetical values refer to sample size, N, for a given year and sample type.

|  | **Percent Success by Sample Year (N)** | | | | | | | | | |
| --- | --- | --- | --- | --- | --- | --- | --- | --- | --- | --- |
| **Sample Type** | *2015* | *2016* | *2017* | *2018* | *2019* | *2020* | *2021* | *2022* | *Unknown* | ***Total*** |
| Whole Blood | 100 (2) | 100 (1) | - | - | 100 (14) | - | - | - | - | **100 (53)** |
| Blood Clot | - | - | 100 (7) | 100 (17) | 100 (9) | 89 (9) | - | - | - | **98 (45)** |
| Serum | - | - | - | 100 (2) | - | - | - | - | 0 (3)^ | **18 (17)** |
| Hair | - | - | - | - | - | - | 94 (17) | 100 (6) | - | **98 (81)** |
| Tissue | - | - | - | 100 (1) | 50 (2) | - | - | - | - | **67 (3)** |
| Cheek Swab | - | 100 (2) | - | - | - | - | - | - | - | **100 (3)** |
| ^The three wolves were present in the population from 1998-2004, 2001-2003, and 2014-2019. Samples were collected for each within their respective time ranges. | | | | | | | | | | |

Table S5: Eighteen nuclear DNA microsatellite loci and 2 sex loci used for gray wolf identification, sibship, and sex confirmation. Loci were dye-labelled and divided into two polymerase-chain reaction (PCR) multiplexes with the listed volumes. Loci were selected from Holmes et al., 1994, Breen et al., 2001, Guyon et al., 2003, Salim et al., 2007, Ostrander et al., 2017 and PCR multiplexes from Clendenin et al., 2020, with modifications to primer input volumes. F+R indicates a mixture of forward and reverse primers.

| **Gray Wolf Multiplex 1** | | | **Gray Wolf Multiplex 2** | | |
| --- | --- | --- | --- | --- | --- |
| *Locus* | *Primer/Reagent* | *1x (μL)* | *Locus* | *Primer/Reagent* | *1x (μL)* |
| FH2670 | 2670 F+R (10μM) | 0.06 | AHT121 | 121F+R (10μM) | 0.07 |
| FH2611 | 2611 F+R (10μM) | 0.07 | C37.172 | 172F+R (10μM) | 0.04 |
| FH2088 | 2088 F+R (10μM) | 0.04 | AHT103 | 103F+R (10μM) | 0.49 |
| FH2054 | 2054 F+R (10μM) | 0.04 | C05.377 | 377F+R (10μM) | 0.04 |
| FH3725 | 3725 F+R (10μM) | 0.08 | AHT109 | 109F+R (10μM) | 0.14 |
| FH2137 | 2137 F+R (10μM) | 0.03 | AHTk200 | 200F+R (10μM) | 0.11 |
| FH2001 | 2001F+R (10μM) | 0.06 | Cxx.250 | 250F+R (10μM) | 0.07 |
| Cxx.119 | CXX119 F+R (10μM) | 0.16 | FH2010 | 2010F+R (10μM) | 0.04 |
| C09.173 | CXX173 F+R (10μM) | 0.04 | - | DNA Extract | 1.00 |
| FH2004 | 2004 F+R (10μM) | 0.11 | - | dH20 | 0.79 |
| DBX6 | DBX6B+DBX61v (10μM) | 0.8 | - | Master Mix (2x) | 3.50 |
| DBY7 | DBY7A+DBY7Iv (10μM) | 0.03 | - | Q-Solution (5x) | 0.70 |
| - | DNA Extract | 2.00 |  |  |  |
| - | Master Mix (2x) | 3.50 |  |  |  |
| - | Q-Solution (5x) | 0.70 |  |  |  |

Table S6: Thermocycler conditions for 2 polymerase-chain reaction multiplexes used for used for gray wolf identification, sibship, and sex confirmation.

| **Gray Wolf Multiplex 1** | | | **Gray Wolf Multiplex 2** | | |
| --- | --- | --- | --- | --- | --- |
| *Step* | *Temperature* | *Time* | *Step* | *Temperature* | *Time* |
| Initial Denature | 94°C | 15 min | Initial Denature | 94°C | 15 min |
| *# of cycles:* | *13* |  | *# of cycles:* | *15* |  |
| Denature: | 94°C | 30 sec | Denature: | 94°C | 30 sec |
| Annealing: | 62°C-0.4°C | 90 sec | Annealing: | 63°C-0.5°C | 90 sec |
| Extension: | 72°C | 1 min | Extension: | 72°C | 1 min |
| *# of cycles:* | *34* |  | *# of cycles:* | *25/30* |  |
| Denature: | 94°C | 30 sec | Denature: | 94°C | 30 sec |
| Annealing: | 57°C | 90 sec | Annealing: | 55°C | 90 sec |
| Extension: | 72°C | 1 min | Extension: | 72°C | 1 min |
| Final Extension: | 60°C | 30 min | Final Extension: | 60°C | 30 min |
| Cooldown: | 4°C | 10 min/∞ | Cooldown: | 4°C | 10 min/∞ |

Table S7: Number of alleles, observed (H_o_), and expected heterozygosity (H_s_) by locus for all individuals (N=381) sampled from Denali National Park and Preserve and Yukon-Charley Rivers National Preserve. Additionally, results by locus are reported for the χ^2^ and exact tests to evaluate Hardy-Weinberg equilibrium. P-values for the χ^2^-test are reported in p-value (χ^2^), whereas p-values from the exact test are under p-value (exact).

| **Locus** | **Alleles** | **H_o_** | **H_s_** | **χ^2^** | **df** | **p-value (χ^2^)** | **p-value (exact)** |
| --- | --- | --- | --- | --- | --- | --- | --- |
| AHT103 | 8 | 0.71 | 0.72 | 17.19 | 28 | 0.94 | 0.82 |
| AHT109 | 6 | 0.68 | 0.71 | 16.39 | 15 | 0.36 | 0.213 |
| AHT121 | 10 | 0.81 | 0.84 | 50.25 | 45 | 0.27 | 0.144 |
| C37.172 | 3 | 0.38 | 0.38 | 0.35 | 3 | 0.95 | 1 |
| AHTk200 | 8 | 0.52 | 0.70 | 113.17 | 28 | 0.00 | 0 |
| FH2004 | 16 | 0.91 | 0.89 | 162.48 | 120 | 0.01 | 0.023 |
| FH2010 | 5 | 0.77 | 0.72 | 25.43 | 10 | 0.00 | 0 |
| Cxx.250 | 7 | 0.61 | 0.69 | 72.39 | 21 | 0.00 | 0 |
| C05.377 | 10 | 0.75 | 0.74 | 91.42 | 45 | 0.00 | 0.005 |
| Cxx.119 | 9 | 0.84 | 0.83 | 41.25 | 36 | 0.25 | 0.166 |
| C09.173 | 6 | 0.74 | 0.77 | 24.23 | 15 | 0.06 | 0.056 |
| FH2001 | 6 | 0.75 | 0.70 | 16.76 | 15 | 0.33 | 0.285 |
| FH2054 | 13 | 0.83 | 0.83 | 65.55 | 78 | 0.84 | 0.134 |
| FH2088 | 6 | 0.68 | 0.67 | 7.97 | 15 | 0.92 | 0.922 |
| FH2137 | 18 | 0.90 | 0.87 | 233.77 | 153 | 0.00 | 0.014 |
| FH2611 | 17 | 0.87 | 0.90 | 160.54 | 136 | 0.07 | 0.014 |
| FH2670 | 16 | 0.79 | 0.82 | 161.12 | 120 | 0.01 | 0 |
| FH3725 | 16 | 0.71 | 0.78 | 225.92 | 120 | 0.00 | 0 |
| *Average* | *-* | *0.74* | *0.75* | - | - | - | - |

Table S8: Number of alleles, observed (H_o_), and expected heterozygosity (H_s_) by locus for all individuals from Denali National Park and Preserve (N=183). Additionally, results by locus are reported for the χ^2^ and exact tests to evaluate Hardy-Weinberg equilibrium. P-values for the χ^2^-test are reported in p-value (χ^2^), whereas p-values from the exact test are under p-value (exact).

| **Locus** | **Alleles** | **H_o_** | **H_s_** | **χ^2^** | **df** | **p-value (χ^2^)** | **p-value (exact)** |
| --- | --- | --- | --- | --- | --- | --- | --- |
| AHT103 | 6 | 0.66 | 0.70 | 68.95 | 15 | 0.00 | 0.105 |
| AHT109 | 5 | 0.64 | 0.69 | 12.47 | 10 | 0.25 | 0.272 |
| AHT121 | 10 | 0.80 | 0.82 | 50.70 | 45 | 0.26 | 0.189 |
| C37.172 | 3 | 0.31 | 0.37 | 4.93 | 3 | 0.18 | 0.058 |
| AHTk200 | 7 | 0.48 | 0.69 | 82.90 | 21 | 0.00 | 0 |
| FH2004 | 14 | 0.92 | 0.88 | 95.73 | 91 | 0.35 | 0.218 |
| FH2010 | 5 | 0.73 | 0.72 | 14.72 | 10 | 0.14 | 0.07 |
| Cxx.250 | 7 | 0.60 | 0.69 | 57.63 | 21 | 0.00 | 0 |
| C05.377 | 10 | 0.72 | 0.72 | 53.77 | 45 | 0.17 | 0.153 |
| Cxx.119 | 9 | 0.83 | 0.83 | 21.06 | 36 | 0.98 | 0.924 |
| C09.173 | 5 | 0.79 | 0.77 | 5.47 | 10 | 0.86 | 0.816 |
| FH2001 | 6 | 0.71 | 0.68 | 9.57 | 15 | 0.85 | 0.656 |
| FH2054 | 11 | 0.80 | 0.80 | 104.66 | 55 | 0.00 | 0.171 |
| FH2088 | 6 | 0.73 | 0.67 | 12.43 | 15 | 0.65 | 0.678 |
| FH2137 | 15 | 0.91 | 0.88 | 172.32 | 105 | 0.00 | 0.023 |
| FH2611 | 13 | 0.85 | 0.90 | 107.25 | 78 | 0.02 | 0 |
| FH2670 | 14 | 0.86 | 0.85 | 96.83 | 91 | 0.32 | 0.008 |
| FH3725 | 13 | 0.69 | 0.78 | 72.01 | 78 | 0.67 | 0.019 |
| *Average* | *-* | *0.72* | *0.75* | *-* | *-* | *-* | *-* |

Table S9: Number of alleles, observed (H_o_), and expected heterozygosity (H_s_) by locus for all individuals from Yukon-Charley Rivers National Preserve (N=198). Additionally, results by locus are reported for the χ^2^ and exact tests to evaluate Hardy-Weinberg equilibrium. P-values for the χ^2^-test are reported in p-value (χ^2^), whereas p-values from the exact test are under p-value (exact).

| **Locus** | **Alleles** | **H_o_** | **H_s_** | **χ^2^** | **df** | **p-value (χ^2^)** | **p-value (exact)** |
| --- | --- | --- | --- | --- | --- | --- | --- |
| AHT103 | 8 | 0.77 | 0.75 | 20.30 | 28 | 0.85 | 0.716 |
| AHT109 | 6 | 0.73 | 0.72 | 24.95 | 15 | 0.05 | 0.026 |
| AHT121 | 10 | 0.83 | 0.86 | 48.45 | 45 | 0.34 | 0.138 |
| C37.172 | 2 | 0.45 | 0.39 | 3.63 | 1 | 0.06 | 0.09 |
| AHTk200 | 6 | 0.56 | 0.70 | 52.22 | 15 | 0.00 | 0 |
| FH2004 | 16 | 0.90 | 0.89 | 156.97 | 120 | 0.01 | 0.168 |
| FH2010 | 5 | 0.82 | 0.72 | 17.05 | 10 | 0.07 | 0.031 |
| Cxx.250 | 6 | 0.62 | 0.69 | 28.81 | 15 | 0.02 | 0.008 |
| C05.377 | 10 | 0.78 | 0.76 | 76.86 | 45 | 0.00 | 0.043 |
| Cxx.119 | 8 | 0.85 | 0.82 | 38.54 | 28 | 0.09 | 0.126 |
| C09.173 | 6 | 0.69 | 0.77 | 30.44 | 15 | 0.01 | 0.002 |
| FH2001 | 6 | 0.79 | 0.72 | 18.05 | 15 | 0.26 | 0.279 |
| FH2054 | 12 | 0.87 | 0.86 | 68.56 | 66 | 0.39 | 0.042 |
| FH2088 | 6 | 0.63 | 0.68 | 15.24 | 15 | 0.43 | 0.461 |
| FH2137 | 17 | 0.89 | 0.87 | 231.27 | 136 | 0.00 | 0.026 |
| FH2611 | 17 | 0.89 | 0.89 | 157.51 | 136 | 0.10 | 0.176 |
| FH2670 | 14 | 0.72 | 0.79 | 109.75 | 91 | 0.09 | 0.001 |
| FH3725 | 15 | 0.73 | 0.78 | 161.06 | 105 | 0.00 | 0.005 |
| *Average* | *-* | *0.75* | *0.76* | *-* | *-* | *-* | *-* |

Table S10: P-values from Fisher’s method to test genotypic linkage disequilibrium in Genepop 4.7.5 (Raymond & Rousset, 1995, Rousset, 2008).

|  | AHT109 | AHT121 | C37.172 | AHTk200 | FH2004 | FH2010 | Cxx.250 | C05.377 | Cxx.119 | C09.173 | FH2001 | FH2054 | FH2088 | FH2137 | FH2611 | FH2670 | FH3725 |
| --- | --- | --- | --- | --- | --- | --- | --- | --- | --- | --- | --- | --- | --- | --- | --- | --- | --- |
| AHT103 | 0.006063 | <4.67e-13 | 0.004258 | 0.146655 | <4.67e-13 | 0.000441 | <4.67e-13 | <4.67e-13 | 0.004554 | <3.99e-07 | 0.00022 | <2.34e-08 | <3.61e-08 | 0.001512 | <2.95e-08 | 0.006448 | <4.57e-08 |
| AHT109 | NA | 0.069774 | 0.092086 | <1.92e-07 | <9.74e-09 | 0.34967 | 0.33333 | <4.67e-13 | <3.42e-10 | 0.002838 | 0.771937 | 0.000196 | 7.24E-06 | <3.31e-07 | <1.81e-08 | 0.016374 | 0.00468 |
| AHT121 |  | NA | 0.054558 | 0.012975 | 0.005935 | 0.003272 | 0.012316 | 0.006848 | 0.000272 | 0.000754 | <4.67e-13 | <1.69e-07 | <7.23e-08 | <1.14e-08 | 0.003498 | <4.67e-13 | 0.002618 |
| C37.172 |  |  | NA | 0.194625 | 0.02877 | 0.111133 | 0.558306 | 0.214929 | 0.001495 | 0.002999 | 0.042119 | 0.142967 | 0.02452 | 0.369535 | <7.10e-07 | 0.006559 | 0.001132 |
| AHTk200 |  |  |  | NA | <1.08e-07 | 0.137831 | 0.039256 | 0.007726 | <9.72e-08 | 0.00824 | 0.08717 | 0.034294 | 0.018531 | <1.66e-09 | <1.19e-07 | 0.00044 | 0.233627 |
| FH2004 |  |  |  |  | NA | <3.08e-08 | <2.95e-07 | 0.002513 | 0.004702 | 0.000722 | 6.44E-05 | <4.67e-13 | <4.67e-13 | 0.000348 | 0.015972 | 0.008597 | <4.67e-13 |
| FH2010 |  |  |  |  |  | NA | <1.20e-07 | 0.010463 | <3.72e-07 | 0.005345 | <1.36e-06 | 0.027335 | <3.60e-08 | 0.000375 | <6.67e-07 | <4.67e-13 | 0.000362 |
| Cxx.250 |  |  |  |  |  |  | NA | 0.002463 | <4.94e-08 | 3.40E-05 | 0.004738 | 0.007442 | 0.038967 | <8.73e-08 | <5.36e-07 | 0.001663 | 0.115526 |
| C05.377 |  |  |  |  |  |  |  | NA | 0.01436 | <5.00e-07 | <1.47e-06 | <3.59e-08 | <4.67e-13 | 0.000556 | 3.45E-05 | <2.69e-07 | <6.49e-09 |
| Cxx.119 |  |  |  |  |  |  |  |  | NA | 0.047296 | 0.237785 | <1.07e-07 | <8.55e-07 | <6.33e-08 | <1.97e-08 | 0.019816 | 0.019608 |
| C09.173 |  |  |  |  |  |  |  |  |  | NA | 0.347188 | <4.43e-08 | <2.12e-07 | 0.053634 | 0.000187 | <2.34e-07 | <2.12e-07 |
| FH2001 |  |  |  |  |  |  |  |  |  |  | NA | 0.000158 | 0.024673 | <2.38e-09 | <5.23e-07 | <1.36e-08 | <9.02e-09 |
| FH2054 |  |  |  |  |  |  |  |  |  |  |  | NA | <4.67e-13 | <4.38e-07 | <5.70e-08 | <5.82e-08 | 0.000455 |
| FH2088 |  |  |  |  |  |  |  |  |  |  |  |  | NA | <2.37e-07 | <4.67e-13 | 0.023373 | <6.65e-09 |
| FH2137 |  |  |  |  |  |  |  |  |  |  |  |  |  | NA | <7.20e-09 | 0.004543 | 0.006843 |
| FH2611 |  |  |  |  |  |  |  |  |  |  |  |  |  |  | NA | <4.67e-13 | <1.38e-08 |
| FH2670 |  |  |  |  |  |  |  |  |  |  |  |  |  |  |  | NA | 0.059129 |

Table S11: Private alleles detected from each study area by locus.

| **Study Area** | **Locus** | **Allele** |
| --- | --- | --- |
| Denali | 172 | 144 |
| Denali | 200 | 220 |
| Denali | 200 | 225 |
| Denali | 250 | 146 |
| Denali | CXX119 | 103 |
| Denali | FH2054 | 180 |
| Denali | FH2137 | 199 |
| Denali | FH2670 | 170 |
| Denali | FH2670 | 222 |
| Denali | FH3725 | 152 |
| Yukon-Charley | 103 | 64 |
| Yukon-Charley | 103 | 82 |
| Yukon-Charley | 109 | 143 |
| Yukon-Charley | 200 | 224 |
| Yukon-Charley | 2004 | 241 |
| Yukon-Charley | 2004 | 253 |
| Yukon-Charley | CXX173 | 112 |
| Yukon-Charley | FH2054 | 164 |
| Yukon-Charley | FH2054 | 179 |
| Yukon-Charley | FH2137 | 175 |
| Yukon-Charley | FH2137 | 179 |
| Yukon-Charley | FH2137 | 201 |
| Yukon-Charley | FH2611 | 186 |
| Yukon-Charley | FH2611 | 200 |
| Yukon-Charley | FH2611 | 218 |
| Yukon-Charley | FH2611 | 222 |
| Yukon-Charley | FH2670 | 214 |
| Yukon-Charley | FH2670 | 218 |
| Yukon-Charley | FH3725 | 162 |
| Yukon-Charley | FH3725 | 168 |
| Yukon-Charley | FH3725 | 196 |

Supplementary Figures

Figure S1: A histogram of the number of wolf samples that lead to successful individual genotypes (n=381) by collection year used in analyses for this paper. Samples collected in Denali are red while samples collected in Yukon-Charley are teal. These counts encompass all sample types, including whole blood, blood clot, serum, hair, tissue, and cheek swabs.


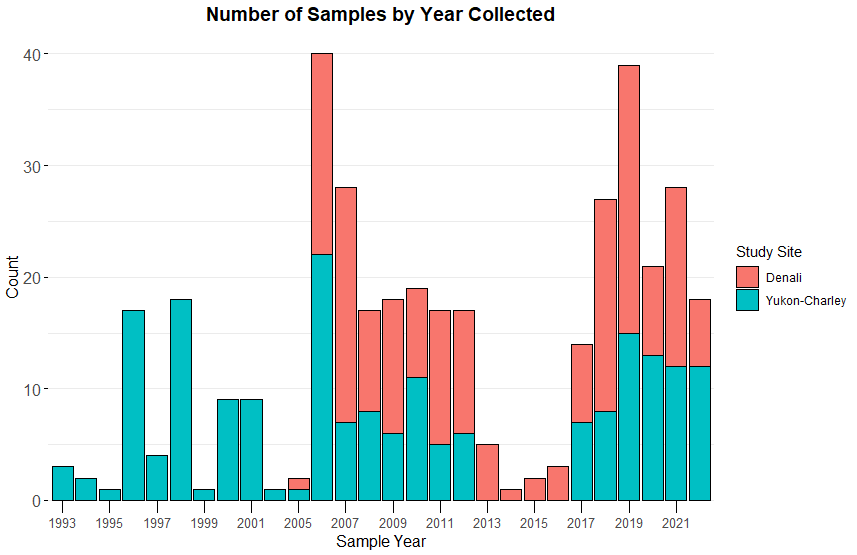


REFERENCES

Breen, M., S. Jouquand, C. Renier, C. S. Mellersh, C. Hitte, N. G. Holmes, A. Chéron, N. Suter, F. Vignaux, A. E. Bristow, et al. (2001). Chromosome-specific single-locus FISH probes allow anchorage of an 1800-marker integrated radiation-hybrid/linkage map of the domestic dog genome to all chromosomes. Genome Res. 11: 1784–1795. doi.org/10.1101/gr.189401

Clendenin, H., J. R. Adams, D. E. Ausband, J. A. Hayden, P. A. Hohenlohe, and L. P. Waits. (2020). Combining harvest and genetics to estimate reproduction in wolves. J. Wildl. Manag. 84, 492-504. doi.org/10.1002/jwmg.21820

Guyon, R., T. D. Lorentzen, C. Hitte, L. Kim, E. Cadieu, H. G. Parker, P. Quignon, J. K. Lowe, C. Renier, B. Gelfenbeyn, et al. (2003). A 1-Mb resolution radiation hybrid map of the canine genome. Proc. Natl. Acad. Sci. U.S.A. 100: 5296–5301. doi.org/10.1073/pnas.0831002100

Holmes, N. G., N. J. Strange, M. M. Binns, C. S. Mellersh, and J. Sampson. (1994). Three polymorphic canine microsatellites. Animal Genetics 25: 200. doi.org/10.1111/j.1365-2052.1994.tb00122.x

Ostrander, E. A., R. K. Wayne, A. H. Freedman, and B. W. Davis. (2017). Demographic history, selection and functional diversity of the canine genome. Nat. Rev. Genet. 18: 705–720. doi.org/10.1038/nrg.2017.67

Raymond M. and F. Rousset. (1995). GENEPOP (version 1.2): population genetics software for exact tests and ecumenicism. J. Heredity 86:248-249. doi.org/10.1093/oxfordjournals.jhered.a111573

Rousset, F. (2008). Genepop'007: a complete reimplementation of the Genepop software for Windows and Linux. Mol. Ecol. Resour. 8: 103-106. doi.org/10.1111/j.1471-8286.2007.01931.x

Salim, D. C., A. A. Akimoto, C. B. Carvalho, S. F. Oliveira, C. K. Grisolia, J. R. Moreira, and M. N. Klautau-Guimarães. (2007). Genetic variability in maned wolf based on heterologous short-tandem repeat markers from domestic dog. Genetics and Molecular Research 6: 348–357.
